# Preparedness and Responses Faced during the COVID-19 Pandemic in Belgium: An Observational Study and Using the National Open Data

**DOI:** 10.3390/ijerph17217985

**Published:** 2020-10-30

**Authors:** Rongxin He, Jun Zhang, Ying Mao, Olivier Degomme, Wei-Hong Zhang

**Affiliations:** 1 International Centre for Reproductive Health (ICRH), Department of Public Health and Primary Care, Ghent University, 9000 Ghent, Belgium; herongxin@stu.xjtu.edu.cn (R.H.); zhangjun08@tsinghua.edu.cn (J.Z.); olivier.degomme@ugent.be (O.D.); 2 School of Public Policy and Administration, Xi’an Jiaotong University, Xi’an 710049, China; mao_ying@mail.xjtu.edu.cn; 3 Research Center for Public Health, Tsinghua University, Beijing 100084, China; 4 School of Public Health, Université libre de Bruxelles, 1050 Bruxelles, Belgium

**Keywords:** public health emergency, preparedness and response, COVID-19, Belgium

## Abstract

This study aimed to descript the Belgian COVID-19 responses process according to the WHO’s (World Health Organization) Health Emergency and Disaster Risk Management Framework (Health EDRM Framework) and to present the measures taken and epidemic impact in the different phases of COVID-19 in Belgium. The WHO’s EDRM Framework was used for reviewing the Belgian Public health emergency preparedness and responses in the context of COVID-19. Information on the measures taken was collected through the literature review including all government’s communication, reports, and scientific papers. All epidemic data were extracted from a national open database managed and published by the Sciensano. Additionally, two authors closely followed the Belgian situation since the beginning of the pandemic and updated the data every day. During the COVID-19 pandemic, the anti-epidemic strategy was mainly to avoid medical resources exceeding the upper limit. Belgium issued a series of emergency decrees to limit the spread of the virus. An existing structure of “federal-region-municipal” as the framework of public health emergency preparedness and response was adapted. The emergency response process in Belgium was divided into four phases: information-evaluation-coordination-decision-making at the region level and the final decision-making at the federal level. Belgium also implemented a phased plan in the process of setting up and lifting the lockdown. However, it was vulnerable in early response, due to the shortage of medical equipment supplies in general, and more particularly for the long term care facilities (LTCFs). Belgium has achieved an intensive cooperation between stakeholders based on an existing multisectoral emergency organization framework. Legislation, medical insurance, and good communication also played a role in limiting the spread of viruses. However, the authorities underestimated the risk of an epidemic and did not take quarantine measures among people suspected affected by SARS-COV-2 in the early stages, resulting in insufficient medical equipment supply and a large number of deaths in the LTCF. The implementation of the lockdown measure in Belgium also encountered obstacles. The lockdown and its exit strategy were both closely related to the pandemic situation and social and economic life. The authorities should strengthen information management, improve the public awareness of the measures, and find out the balance points between the social and economic life and infection control measures.

## 1. Introduction

The outbreak of coronavirus disease 2019 (COVID-19) has spread to 216 countries as of 30 March 2020 and has been officially declared a global pandemic [1]. The World Health Organization (WHO) estimated the pandemic as at 29 July 2020, to be 16,523,815 confirmed cases globally and 655,112 deaths [2]. The epidemic in Europe initially centered around Northern Italy where there was a steep rise in the number of cases and case fatalities from 20 February 2020 onwards [3]. As population density is ranked 4th in Europe [4], the virus was spread across Belgium and the health systems, the economy, and the society was impacted at large. On 4 February 2020, the first case of COVID-19 in Belgium was detected among nine Belgians repatriated from Wuhan, China [5]. Nearly one month later, on 1st March, the second case was reported among travelers returning from Italy [6]. Then the number of confirmed cases increased rapidly as with other western European countries. On 29 March, Belgium reported more than 10,000 confirmed cases [7].

Fortunately, Belgium has a well-functioning healthcare system which is ranked 5th in Europe [8] and has accumulated rich experience in public health emergency response. For example, in 2014, Belgium effectively responded to the importation of the Ebola virus and stopped the spread of the virus in the country [9]. During the COVID-19 pandemic, Belgium implemented a series of measures to mitigate the epidemic impact based on an existing multisectoral response structure. To 20 June, Belgium was reporting the significant reduction of new confirmed cases, hospital admissions, and deaths. The country started phase 3 of the phase-out plan on 8 June [10].

It is important to sum up the experience of the Belgian COVID-19 responses process and share it for the next step to control the pandemic. According to literature search results, there is no research focus on the preparedness and responses faced during the COVID-19 pandemic in Belgium. This study aimed to describe the Belgian COVID-19 responses process according to WHO’s Health Emergency and Disaster Risk Management Framework, and to present the control measures taken and epidemic impact in the different phases of COVID-19 in Belgium.

## 2. Materials and Methods

### 2.1. Analysis Framework

**Figure 1:**
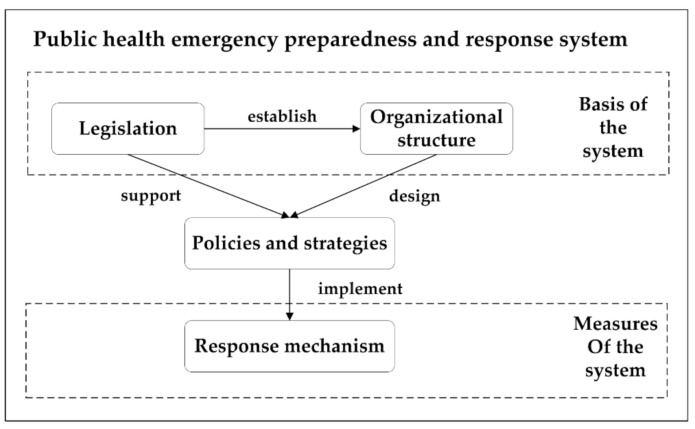
Public health emergency preparedness and response analysis framework.

In 2019, the WHO released the Health EDRM Framework, which clearly defined the objectives, principles, and core components of preparedness and response to public health emergencies [11]. The Health EDRM Framework provides a common language and a comprehensive approach that can be adapted and applied by all actors in health and other sectors who are working to reduce health risks and consequences of emergencies. Based on Health EDRM, a theoretical framework was developed to guide this study. It incorporates three dimensional components (Figure 1). The legislation dimension refers to a series of laws and regulations that support the public health emergency response, while the organizational structure is composed of administrative organizations at all levels. The third dimension of the response mechanism encompasses various measures of the public health emergency response. In this framework, the legislation provides necessary guarantees for the establishment and functioning of the system; organizational structure includes organizations that implement public health emergency policies and strategies; and response mechanisms are the measures in which organizations function within the system.

### 2.2. Data Resource

Information on the measures taken were collected through the literature review including all government communication, reports, and scientific papers. The search strategy was based on a combination of “(COVID-19, or 2019-nCoV), AND emergency, AND (preparedness, response, management) and (Belgium, or Belgian)”. We mainly targeted online media content from the website of government portals, emergency management agencies, and public health agencies in Belgium, including Federal Public Service Public Health, Sciensano (a public health scientific institution), Belgium.be (Official information and services), and Crisis center. All epidemic data were extracted from a national open database managed and published by the Sciensano, a Belgian scientific institution. In addition, two authors closely followed the Belgian situation since the beginning of the pandemic and updated the data every day. This study observed the first three months (from 1 March to 20 June) of the COVID-19 pandemic in Belgium, including the outbreak, peak, declining, and stable phases.

## 3. Result

### 3.1. Preparedness to the COVID-19 Epidemic

Belgium is a country in Western Europe. It covers an area of 30,689 square kilometers and has a population of more than 11.5 million, making it the 4th most densely populated country in Europe [4]. In 2018, more than two million people were aged 65 years and over in Belgium, representing 19% of the country’s population [12]. According to the Belgian Federal Planning Bureau, this proportion will increase to 21% in 2025 and will come close to 26% by 2050 [13]. Currently, there are 815 LTCFs in the Flemish region, 581 in the Walloon region (including the German-speaking regions), and 146 in the Brussels-capital region [14].

Legally, Belgium is a federal constitutional monarchy. Its institutional organization is complex and is structured on both regional and linguistic grounds. It is divided into three highly autonomous regions: the Flemish Region in the north, Wallonia in the south, and the Brussels-Capital Region. The Belgian healthcare system is divided into state and private sectors, with fees payable in both. Federal governments have responsibility for the healthcare system and regional governments are responsible for LTCFs. Both the Belgian federal and regional governments have ministers for public health and a supportive administrative civil service.

#### 3.1.1. Legislation

As early as 1963, Belgium passed the Civil Protection Act. Since then, Belgium has enacted a series of royal or ministerial decrees. In 1988 and 1991, a royal decree established the Government Center for Coordination and Crisis and a Higher Institute for Emergency Planning, respectively [15]. In 2006, a royal decree allowed a step toward harmonizing the terminology and content of emergency plans. To explain the provisions and principles set out in this royal decree, Belgium issued a ministerial decree [16]. In 2009, Belgium issued the other three ministerial decrees, a structure for the general emergency and intervention plan (Plan General Urgency Intervention, PGUI) was available to the provincial governors [17]. In May 2019, the royal decree of emergency planning and management at the municipal and provincial level was issued. The role of mayors and provincial governors in the events and crises requiring coordination or management at the national level was set out [18].

During the COVID-19 pandemic, Belgian legal units oversee the drafting of legal texts and provide answers to the many legal questions that arise in this complex crisis management context. Legal units issued a series of emergency decrees to limit the spread of the COVID-19 (Table 1) [19].

#### 3.1.2. Organizational Structure

**Figure 2:**
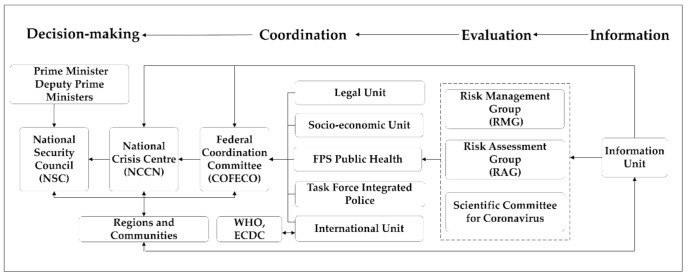
Public health emergency organizational structure. **ECDC:** European Centre for Disease prevention and Control.

The framework of public health emergency preparedness and response in Belgium is a “federal-region-municipal” structure. In the context of COVID-19 preparedness, the following main stakeholders have been involved: National Security Council (NSC), National Crisis Centre (NCCN), Federal Coordination Committee (COFECO), the Ministers-President of the Regions and Communities, FPS Public Health, Risk Assessment Group (RAG), Risk Management Group (RMG), Scientific Committee for Coronavirus, and some other units (Figure 2). NSC essentially consists of the Prime Minister and the Deputy Prime Ministers. COFECO is made up of the Chair of the RMG and representatives of the Prime Minister, the Federal Ministers for Home Affairs, Justice, Finance, Foreign Affairs, Public Health, Budget, Mobility, Defence, Employment, and Labour, as well as the Ministers-President of the Regions and Communities. RAG is chaired by Sciensano (a scientific institution) and comprises experts from Sciensano and the health authorities. RMG is chaired by the FPS Public Health and is made up of representatives of the health authorities, both from the federal state and the federated entities [20].

The emergency response process is divided into four phases: information-evaluation-coordination-decision-making at region level and the final decision-making at the federal level, which not only considers health issues, but also balances the economic, political, social, and cultural aspects of the country. In the information phase, the information unit collected information from regions and communities and gave them feedback. It provided strategic advice to the competent authorities by basing its actions on the identified information needs of the public. In the evaluation phase, public health sectors (RAG, RMG, Scientific Committee) analyzed the public’s risk based on epidemiological and scientific data from regions and communities, and provided scientific advice on the emergency. At the coordination phase, FPS public health and other units in COFECO gave comprehensive advice to NCCN and NSC for preparing and coordinating the implementation of the policy decisions. In the four stages, the regions and communities were fully involved.

### 3.2. Response to the COVID-19 Epidemic

#### 3.2.1. Timeline

**Figure 3:**
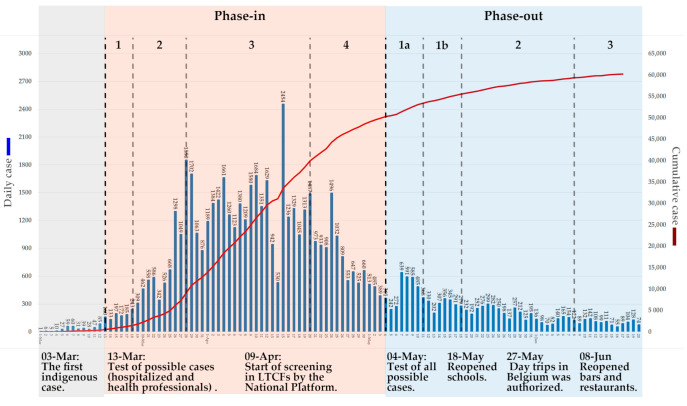
New and cumulative confirmed COVID-19 cases and actions were taken in Belgium.

Over four months, several actions and decisions were taken in Belgium. Figure 3 provides an overview of key dates.

##### Before the Lockdown

After the first case of COVID-19 in Belgium was detected on 4 February, nearly one month later, the second case was found in a traveler returning from Italy on 1 March [5,6]. The authorities required the travelers to pay special attention to their health, but did not take quarantine measures. The first indigenous case occurred on 3 March and the first death was reported on 11 March [21,22]. Meanwhile, the public health sector failed to foresee a large-scale outbreak of coronavirus and did not replenish medical supplies in time.

##### During the Lockdown

With the number of confirmed cases increasing rapidly, the NSC implemented a phase-in lockdown plan based on the advice from public health experts. The phased-in plan consists of four stages.

The first stage (13 March–17 March): As the number of confirmed cases came to 399 and the rate of growth continued to rise, the NSC decided to start the lockdown plan on 13 March by closing schools, restaurants, and cafes, and stopping all kinds of gatherings [23].

The second stage (18 March–27 March): The number of confirmed cases came to 1243 on 17 March, more than three times as many in the early part of the first stage. The confinement and far-reaching social distancing measures were implemented; telecommuting was recommended; ‘non-essential’ stores were closed; and travel was restricted. The police were called out to regulate people’s behavior. Violations of the regulations were subject to heavy fines [24].

The third stage (28 March–19 April): The number of confirmed cases came to 7284 and the number of new cases daily was 1049 on 27 March. The authorities extended the restrictive measures until 19 April and closed the borders. The National Test Platform was established on 5 April, and it implemented a national screening for all LTCFs four days later [25,26].

The fourth stage (20 April–3 May): As the number of daily new cases peaked on 15 April, the NSC extended the restrictive measures until 3 May [27].

##### Lifting the Lockdown

On 6 April, a new expert group was established, the “Group of Experts in charge of the Exit Strategy” (GEES) to prepare for the gradual de-confinement. This group is composed of five scientists (virologists, epidemiologists, biostatistician), three economists (among which is the Governor of the National Bank of Belgium), one jurist, and the Secretary General of the Federation of Social Services [28]. On the basis of the GEES report, the NSC decided to start the phase-out plan as from 4 May [27].

The first stage (4 May–17 May): The number of new cases daily decreased markedly, more patients were discharged from hospitals than admitted. Belgium started the exit strategy: gatherings of up to four people were allowed, at the same time, “contact tracing” was started, people were required to wear mask in public transport, and testing all possible cases began [29].

The second stage (18 May–7 June): The number of new cases daily fell to 279 on 18 May and schools and stores were reopened [30].

The third stage (from 8 June): As the number of new cases daily showed a trend of fluctuating downward during this stage, Belgium started the third phase of the phase-out plan where restaurants, cafes, bars, borders with the Europe or the European Economic Area countries were reopened, and cultural and sporting events were allowed with up to 10 people [31].

#### 3.2.2. Control Strategy

Among all COVID-19 cases, 80–85% of them only showed mild symptoms, and the remaining critically ill cases needed to be admitted to the hospital for treatment [32]. Therefore, the control strategy in Belgium was to avoid health resources exceeding the limit by keeping patients with mild symptoms isolated at home and sending severe patients to the hospital. This strategy used the number of hospital admissions to monitor the population for infections and ensure the health system would not collapse.

**Figure 4:**
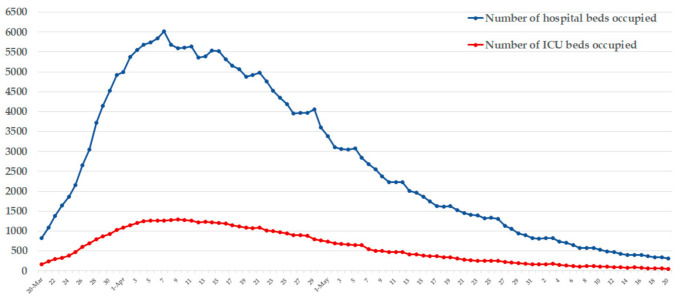
Number of hospital and ICU beds occupied of COVID-19 in Belgium.

Figure 4 provides the number of hospital and the Intensive Care Unit (ICU) beds occupied in the COVID-19 epidemic in Belgium. In early April, the reported number of hospital and ICU beds that were occupied peaked at 6012 and 1285, respectively. However, the ICU bed utilization rate did not exceed 60%, and hospital resources were under control. Then the number of hospital admissions declined steadily [33].

#### 3.2.3. Key Priority Areas and Related Activities

Belgium formulates corresponding emergency plans based on the characteristics of public health emergencies. Based on geographical range and other criteria, the coordination phase of responding to public health emergencies is divided into the municipal phase, regional phase, and federal phase. Corresponding emergency measures are taken in different phases. The implementation of emergency plans or measures includes coordination, communication, resource guarantee, emergency resources, and so on. In the process of Belgian COVID-19 responses, five key priority areas and related activities were refined in this study.

##### Intersectoral Collaboration

The government crisis coordination is handled by the NCCN. And the COFECO is chaired by the NCCN. In the case of COVID-19, the committee prepared and coordinated the implementation of the policy decisions of the NSC at the strategic level. To operationalize these strategic and policy decisions, consultations are organized with the Provincial Governors, the Senior Official of Brussels, and the Minister-President of the Brussels-Capital Region; they, in turn, can coordinate actions at the municipal level [20].

On 12 March, the NSC met in the presence of and in consultation with the Ministers-President. This meeting of the NSC followed on from the meetings of RAG and RMG [23]. The meeting decided Belgium entered the federal phase of crisis management, which means that all decisions were taken by a management team comprised of the Prime Minister, the competent ministers, and the Ministers-President. This phase ensured better coordination and information on the measures taken by the various entities. After that, a series of measures announced applied throughout the country.

##### Testing

There was only one confirmed COVID-19 case in Belgium before March. Therefore, in the early stages, at the slightest suspicion of a possible case of infection, a test was carried out. A total of 128 tests were done before 16 February, for example for the first case from Wuhan was tested ten times [34].

However, as the number of cases increased rapidly, Belgium no longer tested people with mild or asymptomatic cases, but only those admitted to hospital and health workers since 12 March [35]. Since April, Belgium has gradually improved its testing capacity. Due to the rapid increase in the number of deaths reported by LTCF, 20,000 tests were made available for the residents and caregivers in LTCF on 10 April [26]. The daily quantity of PCR tests changed from 2000 in the early stage to 15,430 cases on 19 June. A total of 1,087,369 tests were performed until 19 June, accounting for 9% of the total population [36].

##### Emergency Resources

Emergency resources mainly include financial support, human resources, and emergency supplies. In terms of financial support, the government has invested at least 1 billion euros to respond to the outbreak. NCCN also chaired the information unit, which has a rotation system of 40 people to collect information continuously [20]. The information unit ensures the coordination of all local, regional, community, and federal authorities so that the crisis communication strategies and actions are coherent. It provides strategic advice to the competent authorities by basing its actions on the information of publicly identified needs. NCCN also recruited and trained volunteers as backup power. As for emergency supplies, before the COVID-19 pandemic, 6 million masks were not replenished in time after they expired [37]. That caused Belgium to suffering from a shortage of protective equipment in hospitals. After that, the emergency replenishment of medical supplies was adopted by the government. A large number of supplies were urgently purchased from abroad, and several factories were opened to produce masks; the problem of shortage of materials was gradually alleviated.

##### Risk Communication

The Belgian authorities have continued the information campaigns since the start of the pandemic and kept communicating about the measures needed to stop the spread of the virus. The campaign, which the FPS Chancellery of the Prime Minister was coordinating at a federal level, has come about in collaboration with the FPS Public Health and the National Crisis Centre, in consultation with the communities and regions and with their full cooperation.

The priority was to remind the population at large of the most critical messages. A particular website has been set up to publish the epidemic report every day. Details of the data have been available to the public since 15 March. Other public health messages, such as respecting hygiene measures, respecting social distancing, respecting safety measures, and persevering and staying calm, was disseminated via radio and TV slots, social media, Internet banners, posters or screens in public places, in bus shelters, supermarkets, pharmacies, and on cash machines. All of these messages were in the three national languages of French, Dutch, German, and another thirty languages [38]. Moreover, the messages via radio, print, Internet, or social media adopt the style, colors, and tone of the messaging from the Crisis Centre and the FPS Public Health in order to maintain a coherent and effective approach to all the government’s communication efforts [39].

##### The Vulnerable Populations

The LTCF has been the “severely hit area” of COVID-19 pandemic in Belgium. As with most European countries, Belgium has a large group of ageing population, in which the share of the oldest old (aged 85 and older) is likely to grow even more dramatically from 2.2% in 2010 to almost 3% in 2025 and 5.8% in 2050 [13]. There are approximately 1530 LTCFs in Belgium, with a total of nearly 135,000 beds. The average age of the elderly living there is 85 years old, and 98% of the elderly are aged 75 years and over [14]. The evolution of the COVID-19 pandemic around the world shows that people aged 60 years and over have a higher risk of serious illness after being infected by the virus, especially those who were suffering from serious chronic diseases such as heart disease, diabetes, lung disease, or immune system damage [40,41]. The current statistics on the number of deaths in Belgium include hospital deaths, home deaths, and deaths in LTCF. The data report strategy in Belgium was a transparent and detailed method, even if it resulted in numbers that were overestimated. Until 30 June, 9747 death cases were reported, around 50% of them occurred in an LTCF, and while only 26% of the deaths in an LTCF were confirmed by a test, the other 74% were suspected cases [42]. Therefore, it is not currently possible to confirm that these deaths were caused by COVID-19. Besides, asymptomatic SARS-COV-2 infection was found in Belgian long-term care facilities [43]. Due to the closure of the Office for Foreigners where asylum applications are lodged on 18 March, the first day of the government’s containment measures, new asylum seekers were also effected by receiving government support under the national reception system, which includes housing, medical care, and financial assistance [44].

## 4. Discussion

This study described the Belgian COVID-19 response process and presented the measures taken and epidemic impact in the different phases of COVID-19 in Belgium. We want to discuss and summarize the Belgian experience in this section according to the analysis framework and the WHO’s guidelines, and also the prospects of future studies.

### 4.1. Legislation and Organizational Structure

As we mentioned above, legislative and organizational structures are the basis of the public health emergency management system. They are also the first component highlighted in Health EDRM framework [11]. In the first place, Belgium established a relatively perfect legislation system which defined the structures, roles, and responsibilities of governments and other actors. In the context of the COVID-19 pandemic, Belgium released a series of royal or ministerial decrees and provide legal support for controlling the spread of viruses.

Secondly, Belgium established a fully functional structure, and all key stakeholders felt that the preparedness planning process kept all involved parties engaged. For the organizational structure, efforts at all levels of the public health emergency system (policy-making and operational) were made to prepare and respond to the COVID-19 pandemic. This structure also included a permanent infectious disease risk assessment group with experts in various fields. This group provides scientific advice to authorities to formulate epidemic control measures [20]. However, this organizational structure neglected high-risk institutions such as LTCFs. Belgium introduced prevention regulations to the LTCFs in the early stage of the outbreak, however, severe infection of the staff and the older people of the LTCF resulted due to the lack of strict quarantine measures and the protection equipment being seriously inadequate (masks, protective clothing) [43].

### 4.2. Response Mechanism

The response mechanism is a set of measures of the public health emergency management system, to implement policies and strategies. In the COVID-19 pandemic case, the response mechanism includes collaboration, communication, emergency resource, planning, and information management.

Collaboration: Belgium set up the COFECO, which was chaired by the NCCN and built up intensive cooperation between the health authorities, federal departments, as well as the regions and communities.

Communication: Concise and transparent public and professional communication strategies were implemented, including a multilingual website ensuring the availability of up-to-date information. Multiple ways were adopted to promote public health messages. Belgium also set up the Incident & Crisis Management System (ICMS) which is a modular suite of digital tools to provide the Nation of Belgium with a Federal Incident & Crisis report. It has included 4000 Plus Users at all levels of Government—Municipal, Provincial, and Federal.

Emergency resource: The financial burden of coronavirus testing and treatment are affordable in Belgium. For people who have symptoms of illness and need to be tested, the medical insurance will pay 90% of their testing costs. The medical insurance also covers most of the treatment costs of patients [45]. In the terms of health-care services, all hospitals are prepared for COVID-19 and stopped most of the admission or consultation of non-urgent diseases. That raised the problem of late treatments for patients with other diseases. As to the equipment supply, the national strategic materials encountered problems. The 6 million FFP2 masks in the usual strategic reserve were not replenished in time due to expired destruction. In addition to the global demand for masks, the country experienced a severe shortage of protective items.

Planning (Phased plan): Based on the development of the epidemic and the domestic social and economic situation, Belgium has implemented a phased plan in the process of lockdown and its exit strategy, to ensure that the public health emergency response can be smoothly implemented. The exit strategy is closely related to the pandemic situation and social and economic life. A rush for the exit might lead to a rebound of the pandemic, while too slow will affect social and economic life for a long time. After phase 3 of the exit strategy, there was no significant rebound until the end of June, and the newly confirmed cases were mainly mild and asymptomatic [31].

Information management: The Belgian epidemic data is open, transparent, and comprehensive, and the authorities established a variety of information However, in terms of early warning, the authorities underestimated the risk of an epidemic and did not take quarantine measures among people suspected affected by SARS-COV-2 in the early stages [46], which led to the pandemic spread across the country. Regarding the implementation of the lockdown measures, obstacles were encountered in Belgium, mainly because people did not have enough knowledge about the transmissibility of the coronavirus and were more inclined towards personal freedom. In addition, there a lot of controversy about wearing masks. Belgian people believe that they wear masks only when they are ill. At the same time, the government did not recommend people to wear masks based on the WHO recommendations in the early and middle stages [47]. The above problems reflect that there are still some defects in information management in Belgium.

### 4.3. Strengths and Limitations

To our knowledge, this is the first study focused on the preparedness and responses faced during the COVID-19 pandemic in Belgium. According to the WHO’s Health EDRM Framework, our findings systematically presented an overview of the preparedness and responses process in Belgium from March to June. Limitations also exist in this study. The preparedness and responses process involved a wide range of stakeholders, so it is hard to describe and analyze the actions of each aspect in detail. This study mainly focused on the preparedness and responses faced during the COVID-19 pandemic in federal level. Therefore, it can be concluded that preparedness and responses that took place in municipal or regional levels need to be explored. Future research could also focus on a specific aspect of the emergency preparedness and response system, such as legislation, organizational structure, response mechanism, or more detailed content.

## 5. Conclusions

The anti-epidemic strategy adopted in Belgium was mainly to avoid medical resources exceeding the upper limit, and ultimately achieved the expected results. In this process, Belgium has achieved intensive cooperation between stakeholders established based on an existing multisectoral emergency organization framework, and the health department has provided scientific advice to decision-making through the RAG and RMG. Legislation and medical insurance also played a role for limiting the spread of viruses. Moreover, the Belgian epidemic data is open, transparent and comprehensive, and the authorities established a variety of information communication channels with the public. However, like other European countries, the authorities underestimated the risk of an epidemic, did not take quarantine measures among people suspected of being affected by SARS-COV-2 in the early stages, resulting in an insufficient medical equipment supply and a large number of deaths in the LTCFs. The implementation of the lockdown measures in Belgium also encountered obstacles. The lockdown and its exit strategy are both closely related to the pandemic situation and social and economic life. The authorities should strengthen information management, to improve the public awareness of the measures and to find balance points between the social and economic life and infection control measures.

## Figures and Tables

**Table 1:** Royal or ministerial decrees in Belgium in the context of COVID-19 pandemic.

Date	Contents
17 March 2020	Royal decree concerning the prohibition of supply, putting into service and using rapid self-tests for the measurement or detection of antibodies of SARS-COV-2 virus.
23 March 2020 24 March 2020	Ministerial order carried out emergency measures to limit the spread of the COVID-19.
3 April 2020	Extension of measures to limit the spread of COVID-19 until April 19, 2020.
6 April 2020	Royal decree on the fight against non-compliance with emergency measures to limit the spread of the COVID-19 by the implementation of municipal administrative sanctions.
17 April 2020	Extension of measures to limit the spread of COVID-19.
30 April 2020	The ministerial decree of laying down emergency measures to limit the spread of the COVID-19.
8 May 2020	The ministerial decree updated.
15 May 2020	The ministerial decree updated.
20 May 2020	The ministerial decree updated.
25 May 2020	The ministerial decree updated.
30 June 2020	The ministerial decree updated.

## Abbreviations

COVID-19	Coronavirus Disease 2019
WHO	World Health Organization
EDRM	Health Emergency and Disaster Risk Management
LTCF	Long term care facility
NSC	National Security Council
NCCN	National Crisis Centre
COFECO	Federal Coordination Committee
RAG	Risk Assessment Group
RMG	Risk Management Group
ICU	Intensive Care Unit
GEES	Group of Experts in charge of the Exit Strategy
ICMS	Incident & Crisis Management System
